# Nasopharyngeal Carriage, Antimicrobial Resistance, and Serotype Distribution of *Streptococcus pneumoniae* in Children Under Five in Lebanon: Baseline Data Prior to PCV13 Introduction

**DOI:** 10.3390/antibiotics14020168

**Published:** 2025-02-08

**Authors:** Rayane Rafei, Mazen Zaylaa, Mohamad Diab, Issmat I. Kassem, Khaled El Omari, Fatima B. Halimeh, Grace El Moujaber, Afaf Achour, Bassel Ismail, Hassan Mallat, Monzer Hamze, Fouad Dabboussi, Marwan Osman

**Affiliations:** 1 Laboratoire Microbiologie Santé et Environnement (LMSE), Doctoral School for Science and Technology, Faculty of Public Health, Lebanese University, Tripoli, Lebanon; 2 Faculty of Medicine, Beirut Arab University, Beirut, Lebanon; 3 Center for Food Safety, Department of Food Science and Technology, University of Georgia, Griffin, GA 30223-1797, USA; 4 Faculty of Agricultural and Food Sciences, American University of Beirut, Riad El Solh, Beirut, Lebanon; 5 Quality Control Center Laboratories at the Chamber of Commerce, Industry and Agriculture of Tripoli and North Lebanon, Tripoli, Lebanon; 6 College of Health and Medical Technologies, Alayen Iraqi University (AUIQ), Thi Qar, Iraq; 7 Department of Neurosurgery, Yale University School of Medicine, New Haven, CT 06510, USA

**Keywords:** *Streptococcus pneumoniae*, carriage, children, Lebanon, serotypes, antimicrobial resistance, pneumococcal conjugate vaccine

## Abstract

Background: The nasopharyngeal carriage of *Streptococcus pneumoniae* can be the source of transmission between humans and the starting step towards invasive pneumococcal diseases. Data on the carriage of pneumococci in children before and after the pneumococcal conjugate vaccines (PCV) integration in a country are essential for monitoring any change in pneumococcal carriage serotypes and their antimicrobial-resistance profiles. Methods: We investigated the epidemiology of *S. pneumoniae* carriage among children younger than five years old in Tripoli, Lebanon, in 2016, the same year of integration of PCV13 in the country’s Expanded Program on Immunization. Results: Of 104 participating children, 57 (54.8%) gave a positive culture for *S. pneumoniae*. Antimicrobial susceptibility testing revealed that 26.3% of isolates were multidrug-resistant. Resistance was detected mainly against oxacillin (77.2%), tetracycline (29.8%), erythromycin (22.8%), trimethoprim-sulfamethoxazole (22.8%), clindamycin (19.3%), minocycline (19.3%), and teicoplanin (1.8%). Serotyping analysis identified 14 distinct serotypes, with only 31.3% and 50% of isolates corresponding to vaccine serotypes covered by PCV13 and PCV20, respectively. The most common serotypes were 11A, 19F, 23A, and those of serogroup 24 (Sg24) accounted for 37.5% of the serotyped isolates. Conclusions: Our findings have revealed the circulation of a pool of pneumococci isolates with high levels of antibiotic resistance and different degrees of likelihood of causing invasive diseases in children under five years old in Tripoli in 2016. The overall limited PCV13 vaccine coverage in this study highlighted the need for vaccines with greater coverage in the immunization programs in Lebanon. Longitudinal national studies investigating the carriage of pneumococci in children are required to further assess the impact of the PCV vaccine on pneumococci carriage in children and steer new vaccine development.

## 1. Introduction

*Streptococcus pneumoniae* is an infectious pathogen that causes a wide range of diseases; from mild infections such as sinusitis and otitis to invasive ones such as bacteremia, meningitis, and pneumonia [1,2]. This bacterium is the leading cause of fatal lower respiratory infections globally, accounting for an estimated 653,000 deaths in 2019 [3]. It was also ranked among the five leading bacterial pathogens of global mortality with an estimated 829,000 deaths in 2019 [3]. The human populations that are most vulnerable to pneumococcal diseases include young children, older adults, and immunocompromised individuals [4]. The WHO estimates that *S. pneumoniae* kills more than 300,000 children under five years old yearly [5].

*S. pneumoniae* is also a common commensal of the human nasopharynx and upper respiratory tract [6]. Nasopharyngeal carriage can spread *S. pneumoniae* between hosts and be the starting step towards invasive pneumococcal diseases [1,7,8]. In addition to its wide clinical impact, the mounting resistance in *S. pneumoniae* to antibiotics has led the WHO to stratify penicillin-non-susceptible *S. pneumoniae* within the 2017 priority pathogens list for R&D of new antibiotics [9].

*S. pneumoniae* strains have highly diverse polysaccharide capsule types; with more than 94 serotypes being described to date [10]. The distribution of the serotypes varies geographically, by age, clinical manifestations, and vaccine interventions [11,12]. Currently, the available pneumococcal vaccines, either the pneumococcal polysaccharide vaccines PPSV23 (Pneumovax 23^®^) or the pneumococcal conjugate vaccine PCV (as PCV7 Prevnar^®^, PCV10 Synflorix^®^, PCV13 Prevnar 13^®^, PCV15 Vaxneuvance^®^, PCV20 Prevnar 20^®^), include polysaccharide antigens against a limited number of serotypes that are prevalent in clinical settings [13]. PCV7, a 7-valent formulation, was introduced in 2000 as the first PCV. PCV7 covers 7 serotypes (4, 6B, 9V, 14, 18C, 19F, and 23F) of the 23 serotypes contained in PPSV23. Approximately ten years later, PCV10 and PCV13 were introduced. PCV10 (Synflorix^®^) protects against the PCV7 serotypes and the other three serotypes (1, 5, and 7F), while PCV13 protects against PCV10 serotypes with three additional serotypes (3, 6A, and 19A). In 2021, PCV15 and PCV20 were licensed in the USA and Europe. They are identical to PCV13 with two additional serotypes for PCV15 (22F and 33F) and seven other serotypes for PCV20 (the two PCV15 specific serotypes, 8, 10A, 11A, 12F, and 15B) [14,15]. The introduction of the PCV vaccine has yielded many positive ramifications, including a reduction in both invasive and non-invasive pneumococcal diseases, even among immunosuppressed individuals, as well as a decrease in antibiotic resistance. However, serotype replacement and clonal expansion by serotypes not targeted by the vaccine can potentially mitigate the benefits of PCVs [15,16]; thus, there is an urgent need to follow the trends of vaccine and non-vaccine serotypes.

In Lebanon, PCV was introduced by the private sector. PCV 7 was introduced in 2006, followed by PCV10 and PCV13 in 2010. Since January 2016, PCV13 has been part of the country’s Expanded Program on Immunization [17]. Nevertheless, data on the carriage of pneumococci in the Lebanese population and the circulating serotypes are still missing. Indeed, most published studies investigated the epidemiology of invasive and non-invasive pneumococcal diseases [11,17,18,19,20]. However, studies gauging the epidemiology of pneumococci carriage are essential for understanding population biology and transmission of pneumococci, assessing the impact of available vaccines, and evaluating the performance of new vaccines on a national scale. This study investigates the epidemiology of *S. pneumoniae* carriage among schoolchildren younger than 5 years old in three schools (orphanage [Dar Al Zahra’] and two private schools [Sainte Famille Maronite and Jil Alwa’ed]).

## 2. Results

### 2.1. Streptococcus pneumoniae Carriage

Nasopharyngeal swabs were taken from 104 children, including 45 females and 60 individuals from low socioeconomic status backgrounds. Socioeconomic status was assessed using a questionnaire, with all private school students classified as relatively high-income. In contrast, the parents of the orphanage students face financial difficulties and are unable to meet their children’s basic needs, which is why they place their children in the orphanage to receive free shelter, food, and education. For this reason, we used the type of school as a proxy for distinguishing between high and low socio-economic status. The carriage rate of *S. pneumoniae* was 54.8% (57/104 children) [95% CI: 44.7–64.6%] (Table 1). About 25 females and 32 males were found to be colonized by *S. pneumoniae*. The carriage prevalence in each school was the following: 37.5% (12/32) in Jil Alwa’ed school, 50% (6/12) in Sainte Famille Maronite school, and 65% (39/60) in Dar Al Zahra’ school (orphanage). Hence, the prevalence of carriage in the orphanage and private schools was 65% (39/60) and 40.9% (18/44), respectively.

The statistical analysis did not reveal any association between *S. pneumoniae* carriage and sex distribution or age distribution (*p*-value > 0.05). However, when we stratified students attending private schools as having high socioeconomic status and those in the orphanage as having low socioeconomic status, the multivariable logistic regression found that a low socioeconomic status significantly increased pneumococci carriage (OR: 2.68; 95% CI: 1.21–6.07; *p*-value = 0.016) (Table 2).

### 2.2. Antibiotic Susceptibility of Streptococcus pneumoniae

Of 57 isolates, 80.7% (46 isolates) were resistant to more than one antibiotic and 26.3% (15 isolates; 95% CI: 15.5%−39.7%) were MDR, showing resistance to three or more antibiotic classes. A total of 13 different antibiotic susceptibility patterns were observed, with some isolates demonstrating resistance to up to 6 antibiotics (Table 3). Resistance was detected against 7 out of 13 tested antibiotics: 44 (77.2%; 95% CI: 64.1%−87.3%) isolates were resistant to oxacillin, 17 (29.8%) resistant to tetracycline, 13 (22.8%; 95% CI: 12.7%−35.8%) resistant to erythromycin, 11 (19.3%) resistant to clindamycin, 11 (19.3%) resistant to minocycline, and 1 (1.8%) to teicoplanin. With regards to trimethoprim-sulfamethoxazole, 13 (22.8%) isolates were resistant to this antibiotic, 2 (3.5%) were susceptible to increased exposure (intermediate), and the remaining isolates were susceptible. Out of the 57 isolates, 4 were susceptible to levofloxacin, and the other 53 were susceptible to increased exposure (intermediate) of levofloxacin. No resistance was detected against the other five antibiotics used (norfloxacin, vancomycin, rifampicin, pristinamycin, and linezolid). The 44 isolates resistant to oxacillin were denoted as pneumococci with reduced susceptibility to penicillin.

Among the 13 macrolide-resistant isolates, 11 expressed the cMLSb phenotypes and carried the *ermB* gene, and the remaining 2 had the M phenotype with both the *mefA* and *mefE* genes (Table 3). Of the *ermB*-carrying isolates, two contained the *mefA* gene, and one harbored the *mefA* and *mefE* genes. All 13 macrolide-resistant strains (100%) showed a co-resistance to oxacillin, representing a total of 13 out of 44 strains (29.5%) of all oxacillin-resistant strains (*p*-value = 0.026). In line with the susceptibility of the isolates towards fluoroquinolones (norfloxacin and levofloxacin), pyrosequencing the QRDR of *parC* and *parE* genes in all 57 isolates did not reveal any first-step mutations.

The multivariable logistic regression analysis showed a significant association between erythromycin-resistant *S. pneumoniae* and participants with high socioeconomic status (OR = 0.18; 95% CI = 0.05–0.67; *p*-value = 0.012), but not according to their age or sex (Table 4). Similarly, the same significant link was also noted between MDR *S. pneumoniae* and participants with relatively high socioeconomic status (OR = 0.27; 95% CI = 0.08–0.94; *p*-value = 0.040) (Table 5). Regarding oxacillin-resistant *S. pneumoniae*, no significant association was determined among the participants according to their sex, age, and socioeconomic status (Table 6).

### 2.3. Molecular Serotyping

**Figure 1:**
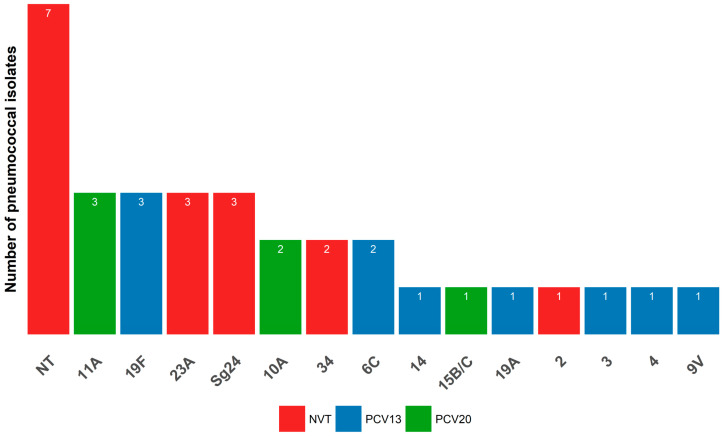
Distribution of serotypes among the *Streptococcus pneumoniae* isolates. NT for a non-typeable isolates; Sg24 for Serogroup 24.

Only 32 isolates were serotyped molecularly by the PCR Takyon kit (Fondation Mérieux, France) due to logistical reasons. Fourteen different serotypes or serogroups were identified in 25 isolates as specified by the kit and the remaining 7 isolates were not assigned to any PCR-targeted serotype (Table 7, Figure 1). The most common serotypes were 11A, 19F, 23A, and those of serogroup 24 (Sg24); each accounting for 9.4% (3/32) of the isolates. Each of the serotypes 34, 10A, and 6C occurred in 6.3%s (2/32) isolates whereas the serotypes 14, 15B/C, 19A, 2, 3, 4, and 9V were of unique distribution and was present in 3.2% (1/32) of isolates. The vaccination coverage of PCV7 and PCV10 against our isolates was 18.8%, for PCV13 and PCV15 was 31.3%, and for PCV20 was 50%.

## 3. Discussion

Although this study was conducted one month after the introduction of the PCV13 vaccine in the Expanded Program of Immunization (with three doses at 4, 6, and 12 months) in January 2016, which made the vaccine available to all children, the study population, aged between three and five years, had not yet benefited from this program. This study, therefore, could give a baseline assessment of the carriage rate, the resistance percentages, and the serotypes circulating in infantile carriers before PCV13 was introduced systematically in the public sector.

The detected carriage prevalence in our sample (54.8%) is close to the pooled prevalence estimate of pneumococcal carriage for healthy children, <5 years of age, of about 64.8% in low-income countries and 47.8% in lower-middle-income countries [24]. Likewise, 56.3% of children under five years in Indonesia were colonized with *S. pneumoniae* before the PCV13 introduction [25]. In Portugal, the prevalence of carriage remained constant (~60%) among children less than 6 years old even after the introduction of PCV13 [26]; a stability not observed elsewhere [27]. In WHO’s Eastern Mediterranean (EMRO) region countries, carriage studies are limited, and five Eastern Mediterranean Region (EMRO) countries (Jordan, Egypt, Somalia, the Syrian Arab Republic, and the Islamic Republic of Iran) have not yet included the PCV in their national schedule as of 2022 [28]. Our prevalence surpassed that found in Egypt (29.2%) [29] and Jordan (33.5%) [30] but was similar to Palestine (55.7%) before the introduction of PCV as part of a Palestinian National Immunization Program [31].

Within the studied sample, the carriage rate among orphanage school children (65%) was significantly higher than that found among private school children (40.9%) (Table 2). This difference could probably be tied to several risk factors among children living in orphanages, including other socioeconomic conditions, crowding [32], and differences in vaccination. Notably, most parents were unaware if their children were PCV vaccinated and only 10% claimed a PCV vaccination according to the parents’ recall. Although the recalling is subjected to bias, it may partly explain such carriage differences between the two examined populations. In Lebanon, before the official incorporation of the pneumococcal vaccine in the immunization routine, PCV was given in the private sector, but no official data existed about the number of pneumococcal-vaccinated children. However, according to the estimations based on drug company sales, approximately 18.5% of children who are 2 years old or younger received an average of three doses of the conjugated pneumococcal vaccine in 2010 and 2011 [20].

High levels of antibiotic resistance were found among isolated strains, with 26.3% being MDR. Nevertheless, some countries, such as Palestine (34.1%) [31], Egypt (41%) [29], and Tunisia (56.6%) [33], communicated higher levels of MDR. Roughly, 77.2% of pneumococci isolates were of reduced susceptibility to penicillin. This percentage seemed relatively high compared to many regions across the world [34] but also comparable to other countries such as France, Jordan, and Kenya before the generalization of vaccines [35,36,37]. Additionally, higher levels of oxacillin resistance (95.3%) were observed in Yemen among asymptomatic children following the implementation of the PCV, although the vaccination was not fully completed [38]. Although the Lebanese studies are often hard-to-compare due to the heterogeneities of adopted breakpoints, susceptibility criteria, and studied periods, our percentage of pneumococci with reduced susceptibility to penicillin is generally higher than those found in clinical isolates [18,20,39,40,41,42,43]. Compared to other age groups, the percentages of invasive isolates resistant to penicillin in Lebanon were the highest in children under 5 years in comparison to the past 15 years; at 25.4% (2005–2009), 23.2% (2010–2015), and 14% (2016–2020) [11]. The richness of antimicrobial resistance in a carriage over invasive isolates has been highlighted in many studies and partly explained by the extent of the genetic exchange among bacteria in the human pharyngeal microbiota [44]. In the same line, a higher occurrence of penicillin-resistant isolates in upper respiratory specimens (31.8%) over lower respiratory isolates (3.7%) was also noted during the first nationwide study in Lebanon that occurred from December 2000 to May 2001, and was partially interpreted by the frequent administration of ß-lactam antibiotics to patients with recurrent upper respiratory infections [43]. Indeed, misconceptions and malpractices associated with antibiotics in childhood upper respiratory tract infections have been documented [45], which can trigger the development of antibiotic resistance in resident microbiota.

Alarmingly, all erythromycin-resistant strains were also non-susceptible to penicillin. The percentage of erythromycin resistance (22.8%) with the predominance of cMLSb phenotype and *ermB* genotype was also consistent with other Lebanese recorded resistance percentages, phenotypes, and genotypes associated with macrolides in clinical isolates [18,20,46,47]. For instance, the percentage of non-susceptibility against erythromycin and clindamycin was 35% and 25%, respectively, when analyzing antibiotic resistance data from 13 hospitals distributed across Lebanon during 2015−2016 [40]. In Arab countries, different percentages of erythromycin resistance were reported in carriage isolates such as 44.2% in Yemen [38], 57−78.2% in Jordan [30], and 30.3% in Palestine [31]. In France, after PCV7 implementation, an immediate reduction in the erythromycin non-susceptibility rate was observed [36]. In 2022, the resistance rate in invasive isolates in France was 30% in children and 23% in adults, with MLSb being the major resistance mechanism [48].

Although our strains were susceptible to quinolones in which corroborated many Lebanese studies with percentages of susceptibility exceeding 98% [18,20], the emergence of levofloxacin-resistant isolates in the Lebanese clinical settings underlines an urgent need for effective measures to curb these resistance trends [11,40]. Resistance against tetracycline in our isolates was 29.8%, a percentage lower than that found in clinically invasive isolates in Lebanon in the PCV7 era (2005−2009: 35.9%) and the post-PCV7/pre-PCV13 era (2010−2015: 34.7%) [11]. Similar findings were obtained in Belgium, where resistance against erythromycin and tetracycline was significantly more frequent in invasive pneumococcal diseases (26.0%; 23.0%) compared to carriage strains (18.2%; 14.5%) [49]. A higher percentage of tetracycline was also recorded in Jordan (45.7%, 51.9%) [30] and Egypt (49%) [29] among carriage isolates. Moreover, the resistance against trimethoprim-sulfamethoxazole among our isolates was 22.8%, which is below the national rate (45%) observed for clinical isolates in Lebanon in 2015/2016 [40], and rates from carriage isolates in Egypt (55%) [29], Jordan (68.6%, 86.6%) [30], and Palestine (45.9%) [31]. In agreement with data from clinical isolates in Lebanon, no resistance against vancomycin was revealed in carriage isolates [11,18]. Meanwhile, a small percentage of resistance observed towards teicoplanin (1.8%) requires further vigilance to avoid any spread of glycopeptide resistance.

In Lebanon, antibiotic misuse is a common issue, stemming from a low level of antibiotic awareness, and improper practices of dispensing antibiotics without a prescription [45,50,51,52]. Approximately half of the Lebanese population self-administer antibiotics [50]. Although studies usually demonstrated an association between antibiotic misuse and parents’ lower educational and socio-economic levels [45], a high socioeconomic status has been found here to be associated significantly with resistance to erythromycin and the MDR pattern of *S. pneumoniae* isolates from the carriage. Our findings may partially reflect that parents with higher economic status have greater access to erythromycin for their families, with or without a prescription. This increased availability could potentially contribute to higher transmission rates and a greater prevalence of erythromycin-resistant *S. pneumoniae*. Again, this highlights an ultimate need to enhance awareness campaigns and implement strict regulations prohibiting selling antibiotics without a prescription. Another potential reason for this association may be the different distributions of serotypes based on socioeconomic status. However, we cannot verify this hypothesis because of the limited number of serotyped isolates and the diversity among circulating serotypes.

The molecular serotyping of 32 isolates unraveled a diverse population of serotypes, where only 31.3% and 50% of isolates were vaccine types included in PCV13 and PCV20, respectively. Interestingly, half of the isolates are considered non-vaccine types. This proportion of vaccine serotypes among pneumococcal isolates was lower than that found in other studies before PCV integration. For example, a study performed in Indonesia before the integration of PCV13 revealed a proportion of about 54.4% of PCV13 serotypes among isolates from children aged <5 years old [25]. A similar percentage of 55.5% was also recorded in Brazil before introducing the PCV10 among children [53]. In the EMRO countries that have not integrated the PCV vaccine yet or have studied the PCV13 coverage before the introduction of PCV in their national programs, the percentage of PCV13 serotypes among carriage isolates was 61.2% among unvaccinated children in Palestine [31], 66.9% in Jordan [30], and 67.4% in Egypt [29]. The low rate of PCV13 observed in our study may be explained by the slight impact of PCV vaccines introduced in the private sector before the public one, which might have reduced the transmission between children and thereby the carriage, even though the estimated national vaccine uptake in the private sector is not high. Indeed, PCV, after its introduction, has remarkably lowered the colonization rate of PCV13 serotypes in many settings [26,54]. An analysis of articles published from 2014 to 2015 revealed a higher prevalence of PCV13 serotypes in invasive diseases than in carriage and non-invasive conditions among children under seven years. This is explained by the greater impact of PCV in reducing nasopharyngeal carriage of PCV-specific serotypes than in invasive disease [12].

The percentages of invasive vaccine-type isolates recovered from children under 5 years old between 2005 and 2020 in Lebanon were 68.6% for PCV13 and 77.3% for PCV20, exceeding our percentages [11]. Although the latter study included some isolates after the introduction of PCV13 in Lebanon [11], its PCV13 vaccine coverage percentage (68.6%) was similar to that (69.3%) obtained in another Lebanese study examining invasive isolates from patients five years old or younger between 2005 and 2011 [20]. The Lebanese authors noted a significant impact of the vaccine introduction on invasive infections caused by PCV7 serotypes but not on PCV13-only serotypes [11]. In addition, a significant increase in non-vaccine serotypes was also observed in invasive infections from 2005 to 2020 in the age group less than 5 years [11].

Although there is no obvious dominant serotype among our typed isolates due to their high serotype diversity and small number, the potentially prevalent serotypes were 11A, 19F, 23A, and Sg24; representing 37.5% of our isolates. In Southeast Asia, the three serotypes commonly encountered in pneumococcal carriage in children younger than five years old were 6A/B, 23F, and 19F before PCV introduction [55], thus only sharing the serotype 19F with our serotypes. For instance, serotype 19F was commonly found among prevalent serotypes in children before PCV integration in national programs in Egypt (19F, 6B, and 6A) [29], Jordan (19F, 14, 6A, 23F, and 6B [30]; 19F, 6A/B, 11A, 19A, 14 and 15B/C [56]), Algeria (6B, 14, 6A, 19F, 23F) [57], and in unvaccinated children in Palestine (6A, 23F, 19F, 6B, 14) [31].

In invasive pneumococcal diseases in Lebanon, the leading serotypes detected in children under 5 years were 14, 19F, 1, and 19A; with serotype 14 being significantly associated with children compared to adults [11]. Beyond the 19F serotype detected in both carriage and invasive diseases, the carriage serotypes found in our study were also observed among invasive pneumococcal diseases in Lebanon across all age groups but with different degrees of prevalence [11]. Such differences in serotypes associated with invasive and carriage contexts are well-documented and shaped by geographical contexts. In Belgium, during the three-year study period (2015−2018), the most commonly detected pneumococcal serotypes in children up to 30 months-old were 23B, 23A, and 11A in carriage, while 12F, 19A, and 10A were in invasive pneumococcal diseases [49]. With regards to 19F, it almost completely disappeared as a serotype involved in pediatric invasive pneumococcal disease after PCV13-introduction in Belgium in 2011, but was still carried at a low level in healthy children [49]. However, 19F and 19A persisted among the dominant serotypes colonizing infants after four years of PCV13 integration after PCV13 introduction in Botswana [21]. The detection of 19F in both contexts (carriage and invasive diseases) in Lebanon is concerning, because it is a common cause of invasive disease in many settings and has a high association with antibiotic resistance [11].

Our findings also raise the question of how likely the 14 serotypes identified here in carriage among children can cause invasive diseases. In general, serotypes 24F and 4 are considered serotypes with high invasive disease potential. Serotypes 14, 19F, 3, 19A, and 10A have medium invasive disease potential, although serotypes 14 and 3 could be stratified within the “high invasive disease potential” group and 19F within the “low invasive disease potential” group [15]. Serotypes 34, 11A, 23A, 6C, 15B/C, 9V, and 24A have low invasive disease potential. Serotype 2 is not classified [15,58], but it has been found to be invasive in South Asia following the PCV10 introduction [59]. Notably, this classification cannot be fully extrapolated to our settings due to different policies and histories of vaccine integration between countries. However, it can be concluded that diverse serotypes with different invasiveness potential circulated in children in Tripoli in the baseline period.

We are unable to identify the serotypes in seven isolates by using multiplex PCR for serotyping, and they were assigned as non-typeable. Similarly, 15.3% of the isolates from carriage could not be serotyped by multiplex PCR in Brazil [53]. Notably, non-typeable isolates were more observed in carriage studies than invasive diseases and their percentages have increased after wide use of PCV in some countries [60,61]. Nevertheless, using additional techniques such as whole genome sequencing or Quellung reaction is advised in the future to verify these observations.

The detection of Sg24, which could contain serotypes 24A, 24B, 24C, and 24F, requires special attention [62]. Among these serotypes, the non-PCV serotype 24F was reported as one of the emerging serotypes in Europe and Western Pacific regions, but not North America [63]. In Lebanon, a significant increase in the prevalence of serotype 24F from 2013 to 2019 was observed in invasive infections with children under 6 years old being the main source of the isolates [17]. In France, serotype 24F was the predominant serotype involved in ~15% of bacteremia in children less than 15 years old in 2022, while it was less represented in nasopharyngeal carriage isolates; suggesting that the 24F serotype is not a colonizing serotype [48,58]. Interestingly, this serotype is one of the notorious serotypes for its antibiotic resistance. For instance, serotype 24F alone represented 35% and 22% of isolates with reduced susceptibility to penicillin that were involved in bacteremia and meningitis, respectively, in children under 15 years old [48]. All three Sg24 isolates in our study had reduced susceptibility to penicillin. Two were erythromycin-resistant with cMLSb phenotype and *ermB* gene, further stressing the MDR phenotype of this serogroup (Table 3). In clinical invasive isolates, serotype 24F was an important contributor to tetracycline resistance during the PCV13 era (2016−2020) in Lebanon [11]. The limited number of isolates in our study precludes unraveling probable associations between serotypes and antibiotic resistance. In addition to Sg24, several identified serotypes herein were commonly associated with antibiotic resistance, such as 14, 19F, 19A, 11A, and 15B/C [11,33,48,64,65].

Although this study is the first report on *S. pneumoniae* carriage in Lebanon, it has some limitations. The lack of data in our sample group on the number of vaccinated people who may have received the PCV vaccine available in the private sector and the type of received vaccines prevented comparisons between vaccinated and unvaccinated people. In addition, the carriage rate was gauged based on culture rather than PCR from the nasopharyngeal specimens, which could also cause an underestimation of the carriage rate [66]. Furthermore, while the oxacillin test can identify isolates with reduced susceptibility to penicillin, it cannot determine the level of resistance to benzylpenicillin or other β-lactams. Therefore, in cases of serious infection or when strains exhibit reduced susceptibility to penicillin (i.e., resistant to oxacillin), it is essential to measure the minimum inhibitory concentration for β-lactams with established therapeutic efficacy against *S. pneumoniae*, such as amoxicillin, cefotaxime, or ceftriaxone. Although the limited number of samples and serotyped isolates cannot provide a comprehensive view of the epidemiology of *S. pneumoniae* and serotype distribution in a carriage context across different socio-economic statuses, this study offers valuable initial insights into *S. pneumoniae* carriage among children at the baseline of PCV13 integration in the public sector.

## 4. Materials and Methods

### 4.1. Study Groups and Bacterial Isolation

**Figure 2:**
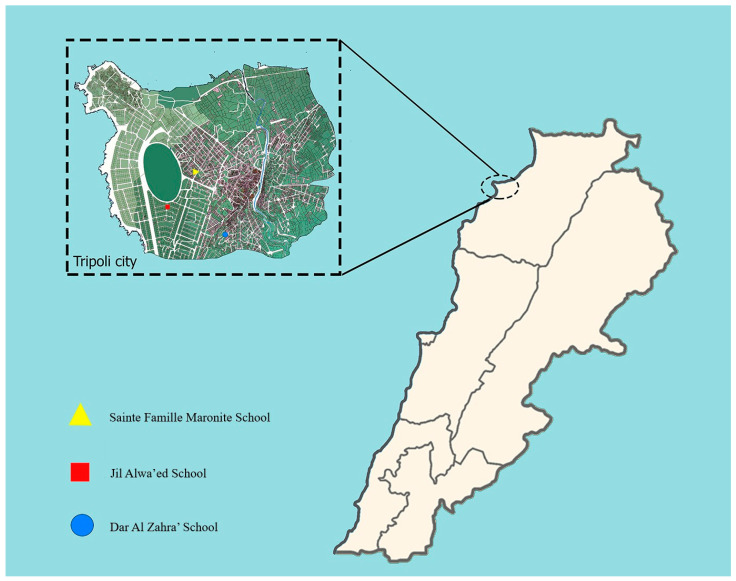
Map of Tripoli city showing the location of schools.

From the beginning of February to the end of May 2016, nasopharyngeal swabs were collected from children aged between three and five years attending three large schools in Tripoli, North of Lebanon: 60 from an orphanage, Dar Al Zahra’, and 44 from 2 private welfare schools (32 from Jil Alwa’ed school and 12 from Sainte Famille Maronite school) (Figure 2). Some differences existed between the orphanage and the two private schools such as the hygienic conditions and socio-cultural and economic levels. Dar Al Zahra’ Orphanage does not only include children who are orphans but also serves as a school for children of low socioeconomic status. This study was approved by the ethical committee of the Doctoral School of Science and Technology at the Lebanese University (approval number CE-EDST-6-2016) and was authorized by the Lebanese Ministry of Public Health. Informed consent was signed by each of the children’s parents or the orphanage administration who also filled out a questionnaire about sociodemographic information.

After the collection of swabs, they were directly transported in Portagerm^®^ medium to the Laboratoire de Microbiologie, Santé et Environnement (LMSE), Lebanese University. Each nasopharyngeal swab was first incubated in BacT/Alert medium (bioMérieux^®^, Craponne, France) with 15 μg/mL of nalidixic acid for 4 h in 5% CO_2_ at 35 °C. After enrichment, around 10 μL of each sample’s inoculum was plated on Columbia CNA plates (BioRad^®^, Marnes-la-Coquette, France) with 5% blood and 5 μg/mL of gentamicin, and then incubated in 5% CO_2_ at 35 °C for 24 h. Preliminary identification of pneumococci was based on presumptive colonies morphology (alpha-hemolytic phenotype), optochin susceptibility (BioRad^®^, Marnes-la-Coquette, France), and positive agglutination latex assay (PASTOREXTM, BioRad^®^, Marnes-la-Coquette, France). A real-time PCR targeting the *lytA* gene was performed as previously described to confirm the pneumococcal identity [67].

### 4.2. Antimicrobial Susceptibility Testing

Antimicrobial susceptibility profiles of *S. pneumoniae* isolates were investigated by the disk diffusion methods on Muller–Hinton agar (Biorad^®^, Marnes-la-Coquette, France) complemented with 5% defibrinated horse blood and 20 mg/L β-NAD. The following antibiotics were targeted: oxacillin (1 μg), vancomycin (5 μg), teicoplanin (30 μg), erythromycin (15 μg), clindamycin (2 μg), pristinamycin (15 μg), rifampicin (5 μg), tetracycline (30 μg), minocycline (30 μg), linezolid (10 μg), norfloxacin (10 μg), levofloxacin (5 μg), and trimethoprim-sulfamethoxazole (1.25 μg/23.75 μg). Antibiotic susceptibility was tested and interpreted according to the recommendations of the CA-SFM (Comité de l’Antibiogramme de la Société Française de Microbiologie; https://www.sfm-microbiologie.org/). A D-test was performed by using erythromycin and clindamycin discs which were placed at a distance of 12 mm apart to detect the phenotypes of macrolide resistance. The cMLSb phenotype (constitutive resistance to macrolide, lincosamide, and type B streptogramin) was identified if the isolate was resistant to erythromycin and clindamycin, while the M phenotype was identified when the isolate was resistant to erythromycin but susceptible to clindamycin without any blunting in the shape of the clindamycin inhibition zone. Oxacillin was used for screening susceptibility toward ß-lactams. If the diameter around the oxacillin was equal to or bigger than 20 mm, isolates were deemed susceptible to ß-lactams tested for *S. pneumoniae*. If the diameter was less than 20 mm, they were considered as pneumococci of reduced susceptibility to penicillin.

Agar plates were then incubated at 35 °C for 24 h in 5% CO_2_. Isolates categorized as susceptible to increased exposure (or intermediate) were considered susceptible. Multi-drug resistant (MDR) isolate was defined as acquired non-susceptibility to at least one drug in three or more antimicrobial classes [68].

Isolates resistant to erythromycin, clindamycin, or pristinamycin were screened for the presence of *ermA*, *ermB*, *ermC*, *linA*, *msrA*, *mefE*, and *mefA* genes as previously described [46]. In addition, 30–40 pb regions of Quinolone Resistance Determining Regions (QRDR) of *parC* and *parE* genes encompassing the well-known mutations contributing to fluoroquinolone resistance were sequenced by pyrosequencing [69].

### 4.3. Molecular Serotyping

The isolates were serotyped by a multiplex real-time PCR assay, which targets 40 serotypes or serogroups previously identified to be most frequently responsible for pneumonia. Eleven PCR reactions were performed on each isolate as previously described [70,71]. The serotypes or serogroups are 1, 2, 3, 4, 5, 6A/B, 6C, 7C, 7F, 8, 9N/L, 9V, 10A, 10F, 11A, 12F, 13, 14, 15A, 15B/C, 16F, 17F, 18C, 19A, 19F, 20, 21, 22F, 23A, 23B, 23F, 24, 31, 33F, 34, 35A, 35B, 35F, 38, and 39, along with an internal control (*lytA*). To calculate the vaccine coverage of PCV7, PCV10, PCV13, PCV15, and PCV20, serotypes 15B/C, and 6C were considered PCV13 serotypes due to the potential cross-protection from PCV 15B serotype antigen to 15C [23] and from the PCV 6A serotype antigen to 6C [21,22]. The percentage was calculated by dividing the sum of isolates with the same serotypes or potential serotypes present in PCV formulations by the total of the isolates. *S. pneumoniae* isolates were classified as non-typeable if the multiplex real-time PCR could not identify a serotype but gave positive results for the internal control *lytA* gene.

### 4.4. Statistical Analysis

The questionnaire data and laboratory results were reviewed for completeness and consistency prior to analysis. Statistical analyses were conducted using R software (R Core Team, version 4.4.0; R Studio, version 2024.04.2–764). A descriptive analysis of all variables was performed with various packages (e.g., dplyr, stringr, prettyR, summarytools), and visualizations were created using the ggplot2 package. Continuous variables were presented as mean ± standard deviation [min–max], while categorical variables were shown as frequency distributions. We predicted the determinants of *S. pneumoniae* nasopharyngeal carriage using univariate and multivariable logistic regression analysis as previously described [72]. Specifically, backward elimination was applied to exclude non-significant factors, optimizing the model based on the lowest Akaike Information Criterion (AIC) score. All statistical tests were two-sided, with a type I error of α = 0.05.

## 5. Conclusions

In this study, we revealed the circulation of a pool of pneumococci isolates with high levels of antibiotic resistance and different degrees of likelihood of causing invasive diseases in children under five years old in Tripoli, North Lebanon in 2016, the same year of the integration of PCV13 in the public sector. Since PCV13 integration, monitoring the changes in pneumococcal carriage serotypes and their antimicrobial profiles has become a pressing need to assess the impact of PCV13 and the usefulness of the new PCV vaccines (PCV15 and PCV20). For instance, some countries such as Portugal have recently witnessed an increase in non-susceptibility to penicillin and erythromycin even after the integration of PCV13 by serotypes not targeted by the current PCV [26]. In Lebanon, at the clinical level, primary analysis has shown a substantial impact of PCVs on invasive pneumococcal diseases and antimicrobial resistance patterns in the population despite an increase in mortality driven by non-vaccine types [11]. Moreover, the limited PCV13 vaccine coverage and the high diversity of carriage serotypes isolated in this study potentially underscore the importance of the inclusion of vaccines (like PCV20) with greater coverage in the national immunization program, as recommended for vaccines for all children younger than 5 years old in the USA [73]. It is also essential to boost the uptake of the already adopted PCV13 vaccine as the rate of vaccinated infants has dropped in the last few years [11]. Finally, national studies investigating the carriage of pneumococci within a long-time frame and larger geographical scale both in children and adults are of utmost importance to track serotype trends and steer the development of new vaccines.

## Figures and Tables

**Table 1:** Demographic characteristics of the study population.

	Dar Al Zahra’	Sainte Famille Maronite	Jil Alwa’ed	Total
**N and (%) of examined individuals**	60 (57.7)	12 (11.5)	32 (30.8)	104 (100)
**N and (%) of females**	23 (38.3)	7 (58.3)	15 (46.9)	45 (43.3)
**N and (%) of males**	37 (61.7)	5 (41.7)	17 (53.1)	59 (56.7)
**Female/male ratio**	0.62	1.40	0.88	0.76
**Mean age ± standard deviation (Min–Max)**	4.02 ± 0.13 (4−5)	4.67 ± 0.52 (4−5)	4.35 ± 0.80 (3−5)	4.16 ± 0.51 (3−5)
**N and (%) of *Streptococcus pneumoniae* carriage**	39 (65.0)	6 (50.0)	12 (37.5)	57 (54.8)

**Table 2:** Distribution of *Streptococcus pneumoniae* nasopharyngeal carriage among the participants according to their sex, age, and socioeconomic status.

		Descriptive Analysis		Fisher’s Exact Test	Multivariable Logistic Regression Analysis		
		Total	Carriage N (%)	*p*-Value	adj. OR	95% CI	*p*-Value
**Sex**	Male ^1^	59	32 (54.2)				
	Female	45	25 (55.6)	1.00			
**Age**	3	6	3 (50.0)				
	4 ^1^	69	44 (63.8)				
	5	22	10 (45.5)	0.266			
**Socioeconomic status**	High ^1^	44	18 (40.9)				
	Low	60	39 (65.0)	**0.018**	**2.68**	**1.21–6.07**	**0.016**

Only variables tested by univariate analysis that had a *p*-value < 0.20 were included in the multivariable logistic regression analysis. Bold and red values indicate statistically significant results. ^1^ Reference group.

**Table 3:** Distribution of resistance profiles among the 57 isolates.

No. of Antibiotics to Which the Isolates Were Resistant Against	No. (%) of Pneumococci Isolates ^a^	Antiobitic Resistance Profile of the Isolates ^b^	No. of Antibiotic Classes	Serotypes ^c^	Macrolide Resistance Genotype
0	11	NA	NA	NT ^d^ (2), 11A (1), 23A (1), 3 (1), 4 (1), 6C (1)	-
1	23	OXA	1	NT (3), 19F (3), 23A (2), 15BC (1), 24 (1), 19A (1), 34 (1)	-
1	1	SXT	1	34 (1)	-
2	4	OXA, SXT	2	10A (2), 11A (2)	-
2	1	OXA, TET	2	-	-
2	1	SXT, TET	2	-	-
2	1	OXA, ERY	2	NT (1)	M phenotype; *mefA*-*mefE*
4	3 ^e^	OXA, TET, MNO, SXT	3	2 (1), NT (1)	-
4	1 ^e^	OXA, ERY, CLN, TET	4	-	cMLSb ^f^; *ermB*
5	6 ^e^	OXA, ERY, CLN, TET, MNO	4	14 (1), 6C (1), 24 (1)	cMLSb; *ermB*
5	3 ^e^	OXA, ERY, CLN, TET, SXT	5	-	cMLSb; *ermB-mefA* (2), *ermB-mefA-mefE* (1)
5	1 ^e^	OXA, ERY, TET, MNO, SXT	4	9V (1)	M phenotype; *mefA*-*mefE*
6	1 ^e^	OXA, ERY, CLN, TET, MNO, TEC	5	24 (1)	cMLSb; *ermB*

^a^ The total number of isolates is 57 isolates. ^b^ NA not applicable, OXA for oxacillin, SXT for trimethoprim-sulfamethoxazole, TET for tetracycline, MNO for minocycline, ERY for erythromycin, CLN for clindamycin, TEC for teicoplanin. ^c^ The total number of tested isolates is 32 isolates. ^d^ NT for a non-typeable isolate. ^e^ These isolates are defined as MDR (Multidrug resistant). ^f^ cMLSb for constitutive resistance to macrolide, lincosamide, and type B streptogramin.

**Table 4:** Distribution of erythromycin-resistant *Streptococcus pneumoniae* among the participants according to their sex, age, and socioeconomic status.

		Descriptive Analysis		Fisher’s Exact Test	Multivariable Logistic Regression Analysis		
		Total	Resistance to Erythromycin N (%)	*p*-Value	adj. OR	95% CI	*p*-Value
**Sex**	Male ^1^	32	7 (21.9)				
	Female	25	6 (24.0)	1.00			
**Age**	3	3	1 (33.3)				
	4 ^1^	44	8 (18.2)				
	5	10	4 (40.0)	0.255			
**Socioeconomic status**	High ^1^	18	8 (44.4)				
	Low	39	5 (11.4)	**0.015**	**0.18**	**0.05–0.67**	**0.012**

Only variables tested by univariate analysis that had a *p*-value < 0.20 were included in the multivariable logistic regression analysis. Bold and red values indicate statistically significant results. ^1^ Reference group.

**Table 5:** Distribution of multidrug-resistant *Streptococcus pneumoniae* among the participants according to their sex, age, and socioeconomic status.

		Descriptive Analysis		Fisher’s Exact Test	Multivariable Logistic Regression Analysis		
		Total	Multidrug Resistance N (%)	*p*-Value	adj. OR	95% CI	*p*-Value
**Sex**	Male ^1^	32	9 (28.1)				
	Female	25	6 (24.0)	0.771			
**Age**	3	3	1 (33.3)				
	4 ^1^	44	10 (22.7)				
	5	10	4 (40.0)	0.452			
**Socioeconomic status**	High ^1^	18	8 (44.4)				
	Low	39	7 (17.9)	0.052	**0.27**	**0.08–0.94**	**0.040**

Only variables tested by univariate analysis that had a *p*-value < 0.20 were included in the multivariable logistic regression analysis. Bold and red values indicate statistically significant results. ^1^ Reference group.

**Table 6:** Distribution of oxacillin-resistant *Streptococcus pneumoniae* among the participants according to their sex, age, and socioeconomic status.

		Descriptive Analysis		Fisher’s Exact Test	Multivariable Logistic Regression Analysis		
		Total	Resistance to Oxacillin N (%)	*p*-Value	adj. OR	95% CI	*p*-Value
**Sex**	Male ^1^	32	26 (81.3)				
	Female	25	18 (72.0)	0.528			
**Age**	3	3	2 (66.7)				
	4 ^1^	44	34 (77.3)				
	5	10	8 (80.0)	0.859			
**Socioeconomic status**	High ^1^	18	14 (77.8)				
	Low	39	30 (76.9)	1.00			

Only variables tested by univariate analysis that had a *p*-value < 0.20 were included in the multivariable logistic regression analysis. ^1^ Reference group.

**Table 7:** Distribution of serotypes among 32 tested isolates along with their susceptibility toward penicillin and erythromycin.

Serotype	PCV Serotype ^a^	No. of Isolates	Susceptibility to Penicillin	Susceptibility to Erythromycin
19F	PCV13 ^a^	3	Reduced (3/3)	S
11A	PCV20 ^b^	3	Reduced (2/3)	S
23A	NVT ^c^	3	Reduced (2/3)	S
Sg24 ^d^	NVT	3	Reduced (3/3)	R (2/3, cMLSb ^e^)
10A	PCV20	2	Reduced (2/2)	S
34	NVT	2	Reduced (1/2)	S
6C ^f^	PCV13	2	Reduced (1/2)	R (1/2, cMLSb)
14	PCV13	1	Reduced	R (cMLSb)
15B/C ^f^	PCV20	1	Reduced	S
19A	PCV13	1	Reduced	S
2	NVT	1	Reduced	R
3	PCV13	1	S	S
4	PCV13	1	S	S
9V	PCV13	1	Reduced	R (M phenotype)
NT ^g^	-	7	Reduced (5/7)	R (1/7, M phenotype)

^a^ PCV13 includes serotypes: 1, 3, 4, 5, 6A, 6B, 7F, 9V, 14, 18C, 19A, 19F, and 23F. ^b^ PCV20 means, here, only the seven additional serotypes covered by the PCV20, but not the PCV13: 8, 10A, 11A, 12F, 15B, 22F and 33F. ^c^ NVT for non-PCV20 vaccine types. ^d^ Sg for serogroup 24 which contains many serotypes. ^e^ cMLSb for constitutive resistance to macrolide, lincosamide, and type B streptogramin. ^f^ Serotype 6C was considered as a PCV13 serotype due to cross-protection from the serotype 6A antigen to 6C [21,22]. The same applies between the serotypes 15 B/C and the PCV 15 B serotype [23]. ^g^ NT for non-typeable isolates.

## Data Availability

The raw data R codes necessary to replicate the analysis are publicly available (DOI: 10.5281/zenodo.13748430).
